# Nanoduct Sweat Conductivity Measurements in 2664 Patients: Relationship to Age, Arterial Blood Gas, Serum Electrolyte Profiles and Clinical Diagnosis

**DOI:** 10.4021/jocmr1191w

**Published:** 2013-01-11

**Authors:** Rabia Gonul Sezer, Gokhan Aydemir, Abdullah Baris Akcan, Cem Paketci, Abdulbaki Karaoglu, Secil Aydinoz, Abdulkadir Bozaykut

**Affiliations:** a Department of Pediatrics, Zeynep Kamil Maternity and Childrens Diseases Training and Research State Hospital, Istanbul, Turkey; b Department of Pediatrics, GATA, Haydarpasa Teaching Hospital, Istanbul, Turkey; c Department of Pediatrics, GATA, Ankara, Turkey

**Keywords:** Cystic fibrosis, Blood gases, Electrolytes, Serum

## Abstract

**Background:**

The Nanoduct^®^ device has acceptable diagnostic accuracy, but there is not enough systematic data supporting its usage in the diagnosis of cystic fibrosis (CF).

**Methods:**

A retrospective review of patients with an indication for the sweat test was conducted. The conductivity test was repeated in patients who had values higher than 60 mmol/L, and they were referred for sweat chloride measurements. Associations between sweat conductivity measurements and age, gender, (pH, HCO_3_, pCO_2_, Na, K, Cl), family history, consanguinity, indications for the test and number of hospitalization were studied.

**Results:**

Among 2,664 patients, 16 children had sweat conductivity values higher than 80. The median age of patients diagnosed with CF was 4 months old. Age, pH, HCO_3_, Na, Cl, K and the sweat conductivity test were statistically related (P < 0.001). The ROC curve showed very high agreement between the 2nd conductivity test and the sweat test.

**Conclusions:**

Patients suspected to have CF can be screened using the Nanoduct^®^ conductivity device in non-qualified centers.

## Introduction

The sweat test is the key method of investigation for the diagnosis of cystic fibrosis (CF) [1]. Conductivity is easier to perform than the classical Gibson and Cook method and requires a smaller amount of sweat [1]. The CF Foundation has approved the use of the Wescor Macroduct Sweat-Check conductivity analyzer for screening purposes at community hospitals [2]. Several studies have shown that conductivity haves an equal potential to discriminate between CF and non-CF patients [3-6]. Conflicting results about the correct diagnostic success rate of the Nanoduct^®^ method have been reported [5, 7]. Although the Nanoduct^®^ device has acceptable levels of diagnostic accuracy, there is still not enough systematic data to support its usage in the diagnosis of CF [8, 9]. Sweat conductivity has been shown to be approximately 15 mmol/L higher than the sweat chloride concentration [4].

The main indications for the sweat test include a phenotype suggestive of CF, family history of CF, follow-up of newborn screening tests and the suspicion of an atypical CF phenotype [1]. The precise prevalence of CF in Turkey is unknown. However, in CF, the high heterogeneity and significantly higher number of mutations compared to other European countries has been demonstrated in several studies [10, 11]. There is no newborn screening program for CF in Turkey at the present time; thus, the diagnosis is made when clinical symptoms exist and the patients’ history is suspicious. This leads to the late diagnosis of CF, with a mean age of 31.2 ± 41.4 (range: 1.5 - 168) months [12]. The early diagnosis of CF is important because the disease becomes chronic in children with a current median predicted age of survival of 37.4 years [13].

In this report, we document our experience comparing sweat conductivity results with metabolic alkalosis and hypoelectrolytemia in patients with a diagnosis of possible CF from 2006 - 2010. The aim of this retrospective study was to report our Nanoduct conductivity results. In addition, we investigated the relationship between the presence of metabolic alkalosis, hypoelectrolytemia and the clinical diagnosis.

## Material and Method

We retrospectively reviewed the medical records of all children who had sweat conductivity tests from July 2006 to November 2010. A total of 2,664 patients were referred for sweat analysis. Few patients had repeated sweat tests with subsequent visits, and the intervals between the tests were less than one month. The study population included patients attending pediatric and pediatric surgery outpatient clinics, and it also included those admitted to the neonatal intensive care unit, pediatric surgery and pediatric medicine wards. There were no patients with known CF. Our hospital was the only training and research state hospital with the facility for sweat conductivity testing at the time of the study, and it was located in the Asian site of Istanbul. Sweat testing was available 5 days per week.

The patients’ clinical history and laboratory data were reviewed from the hospital database computer system. A physical examination was conducted for all patients and further investigations were conducted as needed on a clinical basis. For each patient, age, gender, diagnosis and hospitalization number (if hospitalized) were known. Information regarding serum pH, pCO_2_, HCO_3_, Na, Cl and K values were recorded when available. In many cases, a family history of atopy, allergy, CF, asthma, tuberculosis, presence of consanguineous marriage and the duration of symptoms were also recorded.

Metabolic alkalosis was defined as blood pH levels greater than7.45 and/or serum bicarbonate levels higher than 28 mmol/L in the absence of respiratory insufficiency (PaCO_2_ ≥ 45 mmHg). Hypoelectrolytemia was considered if serum sodium, potassium and chloride were under 135, 3.5, and 90 mEq/L levels, respectively.

The Nanoduct^®^ Neonatal Sweat Analysis System (Wescor Inc., Logan, Utah, Biomedical Products Division) was used for CF sweat test screening. The device was suitable for children under 16 years old, including neonates. Sweat was induced by pilocarpine iontophoresis, and the results were interpreted by continuous-flow analysis on the analyzer display. The flexor aspect of the forearm, approximately halfway between the wrist and elbow, was used for the anodic (positive) skin site. The area was cleaned with alcohol. The results were considered normal if values were between 3 - 60 mmol/L (equivalent NaCl) and intermediate if values were between 61 - 80 mmol/L. CF was very likely if the results were equal to or above 80 mmol/L based on the user’s manual. The minimum reading value was 3 mmol/L. Tests were conducted at room temperature (22 - 25 °C) with a relative humidity of approximately 60%

The same laboratory technician who was specifically educated on the use of this device performed the sweat analysis tests on all of the children. Test results were excluded from the study if not enough sweat was produced for analysis. Other than mild skin erythema, no adverse effects from pilocarpine iontophoresis were reported.

The conductivity test was repeated in patients who had values higher than 60 mmol/L and those with highly suspicious clinical symptoms with normal conductivity results. Values over 60 mmol/L in the second test were referred for sweat chloride measurements because CF should not be diagnosed based on conductivity measurements alone [1]. Twenty patients with high conductivity values (> 60 mmol/L) in the second test and clinical manifestations of CF were referred to the pediatric chest clinic for follow-up. The diagnosis was confirmed in 17 patients with sweat tests, and mutations of the CF transmembrane conductance regulator gene were found in all of them [10]. Three patients did not have CF. Patients with normal and/or borderline values in the second conductivity test were followed to sufficiently eliminate the diagnosis of CF on a clinical basis.

### Clinical material

The following 7 groups were studied: 1). Upper respiratory track diseases: including sinusitis, polyp and non-specific cough; 2). Lower respiratory tract diseases: subacute, recurrent or chronic chest diseases including asthma, bronchiolitis, bronchitis, pneumonia, bronchiectasis, and dyspnea; 3). Gastrointestinal tract diseases: gastroenteritis, malabsorption diseases such as celiac disease, steatorrhoea, pancreatic insufficiency, and liver disease; 4). Delay of passing stool: volvulus, meconium ileus, ileal atresia, Hirschsprung disease, rectal prolapse, constipation, and abdominal distention; 5). Failure to thrive and protein-energy malnutrition; 6). Allergy classified as allergic rhinitis and any documented allergy; 7). Suspicion of CF: atypical symptoms suggestive of CF or children referred from other institutions for sweat analysis.

The study was approved by the ethical committee of Zeynep Kamil Maternity and Children’s Diseases Training and Research State Hospital.

### Statistical analysis

The parametric data were analyzed using paired t-tests. Age groups and conductivity result groups were analyzed by analysis of variance (ANOVA) (SPSS; release 17). The results are presented as the mean ± standard deviation (SD) and the median (minimum-maximum). The association between the two consecutive conductivity tests was assessed by Pearson correlation coefficients, and non-parametric correlations were assessed using Spearman’s correlation coefficients.

The capability of the conductivity test to discriminate between CF and non-CF subjects was assessed by constructing a receiver operating characteristic (ROC) curve, and the best cut-off values were assessed through the calculations of sensitivity and specificity. A P-value of less than 0.05 was regarded as statistically significant.

## Results

### Patient characteristics

Two thousand six hundred and sixty-four patients with an indication for the sweat test were reviewed in this study. The median age was 17 months with a range of 7 days to 17 years old. There were 1,600 (60.1%) boys, 896 girls and 68 newborns included in the study.

There were 1,768 (66.4%) patients from the outpatient clinics, and the remaining 896 patients were hospitalized.

The median age of the patients diagnosed with CF was 4 months (mean ± SD: 13.8 ± 23.7; minimum: 1-month; maximum: 84-months). Twelve out of 17 CF patients were hospitalized at the time of diagnosis, and 5 of them were from outpatient clinics. Eight of the patients had a history of multiple hospitalizations before being diagnosed with CF. The median duration of the patients’ symptoms was 8.5 days, with a maximum value of 90 days.

### Indications for sweat test

The most common indication for performing a sweat test was lower respiratory tract manifestation in 1909 patients (71.7%). Children with recurrent bouts of pneumonia and bronchiolitis comprised the first indication for the sweat test.

The other indications, in descending order of frequency, included non-specific symptoms suggestive of CF (185 patients), upper respiratory tract diseases (184 patients), failure to thrive (155 patients), gastrointestinal disorders (85 patients), delay of passing stool (78 patients) and allergy (68 patients).

The most common presenting manifestation among CF patients was lower respiratory tract disorders, as evidenced in 11 patients (64.7%). Two patients presented with meconium ileus (11.8%), and two patients presented with upper respiratory tract manifestations. Gastrointestinal symptoms (1 patient, jaundice) and a failure to thrive (1 patient) were the other presenting manifestations in patients diagnosed with CF.

### Sweat conductivity test results

From the 2,664 patients included in the study, 16 children had sweat conductivity values higher than 80. There were 76 children who had a second conductivity test, and 13 of them had sweat conductivity values above 80 for the second test (Table 1). The median conductivity values of patients diagnosed with CF in the consecutive two tests were 91 (mean ± SD: 88.2 ± 18.7; minimum: 54; maximum: 119 mmol/L) and 85 (mean ± SD: 84 ± 11.2; minimum: 65; maximum: 99 mmol/L), respectively.

**Table 1:** The Distribution of the Number of Patients was Based on the 1st and 2nd Conductivity Results, and the Numbers in Parantheses Represent the Cystic Fibrosis Patients Confirmed by the Sweat Chloride Measurements

			2nd conductivity test (mmol/L)		
			3 - 60 Normal	61 - 80 Intermediate	> 80 Positive
	3 - 60 Normal	2,588	35	0	1 (1 CF)
1st conductivity test (mmol/L)	61 - 80 Intermediate	0	17	6 (4 CF)	1 (1 CF)
	> 80 Positive	0	4	1	11 (11 CF)

There were no significant differences between the results from boys and girls (P > 0.05). Age, pH, HCO3, Na, Cl, K and sweat conductivity test results were statistically related (P < 0.001). Measurements were significantly higher in children older than 2 years (Table 2).

**Table 2:** The Conductivity Measurements in Patients According to Age Groups

		All Ages (n)	0 - 6 months		7 - 12 months		13 - 24 months		> 24 months	
			Male (n)	Female (n)	Male (n)	Female (n)	Male (n)	Female (n)	Male (n)	Female (n)
	Mean ± SD	34.26 ± 10.34	34.58 ± 14.34	34.79 ± 11.47	32.86 ± 9.83	32.18 ± 8.44	33.47 ± 8.75	33.33 ± 8.14	35.38 ± 10.07	35.61 ± 9.15
1st conductivity test, mmol/L		(2,664)	(366)	(206)	(337)	(204)	(363)	(227)	(534)	(427)
	Min - Max	5 - 131	12 - 131	10 - 91	5 - 110	11 - 62	9 - 80	12 - 58	7 - 106	6 - 77
	Mean ± SD	48.74 ± 22.55	52.41 ± 24.54	45.86 ± 23.11	32.86 ± 9.83	27.67 ± 16.56	44.88 ± 19.08		54.80 ± 23.05	41.12 ± 7.64
2nd conductivity test, mmol/L		(76)	(29)	(14)	(4)	(3)	(8)	(0)	(10)	(8)
	Min - max	12 - 99	17 - 98	12 - 87	31 - 99	12 - 45	24 - 84		31 - 98	32 - 51

**Figure 1:**
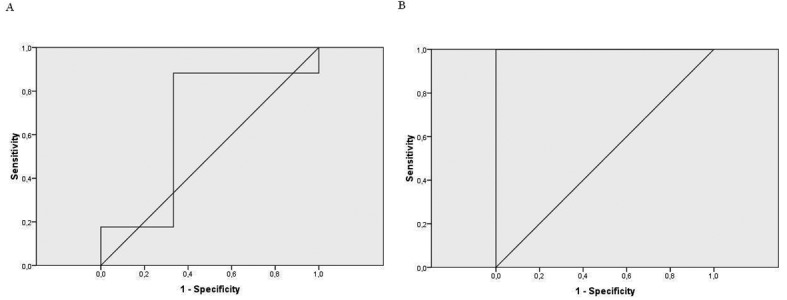
A: the ROC curve for the 1st conductivity test; B: the ROC curve for the 2nd conductivity test.

The Pearson’s correlation between the first and second conductivity test was significant (r = 0.76, P < 0.001). When we correlated sweat chloride results with the first conductivity test, it was not significant(r = 0.18, P > 0.05). However, the second conductivity test correlation was statistically significant (r = 0.62, P = 0.003). The ROC curve showed a very high level of agreement between the second conductivity test and the sweat test (Fig. 1) (Table 3).

**Table 3:** Different Conductivity Cut-off Values of the 1st and 2nd Tests to Predict the Cystic Fibrosis Diagnosis, According to the Number of Patients Confirmed by Sweat Chloride Measurements

		Sensitivity (%)	Specificity (%)	Area Under Cure
1st conductivity test	53 mmol/L	100	100	0.647 P > 0.05
	58 mmol/L	94	100	
	63 mmol/L	88	100	
2nd conductivity test	60 mmol/L	100	100	1 P < 0.007
	61.5 mmol/L	100	66	

### Arterial blood gas and electrolytes

Mean ± SD, median, minimum and maximum values of laboratory parameters among patients with available data are shown based on sweat conductivity results (Table 4). pH, HCO3, Na, Cl and K were statistically significant based on sweat conductivity results (P < 0.05). The significant differences between the pH, HCO3 and Na levels and the conductivity results of the three groups were based on the higher conductivity level (> 80 mmol/L) group. When we compared the Cl values of the patients based on conductivity results, all three groups of conductivity results were significantly different than each other. Data from the patients diagnosed as CF are also given in Table 4.

**Table 4:** The Distribution of Laboratory Values Based on Conductivity Results and the Results of the Patients Diagnosed With Cystic Fibrosis. A Comparison of laboratory Values Based on Normal, Intermediate and Positive Conductivity are Also Given (ANOVA)

		3 - 60 mmol/L	61 - 80 mmol/L	> 80 mmol/L	CF Patients	ANOVA	
		Normal	Intermediate	Positive			
		(n: 509)	(n: 9)	(n: 7)	(n: 17)	F	P
Na	Mean ± SD	138.42 ± 3.75	137.72 ± 3.80	130.21 ± 5.64	133.43 ± 6.2	17.1	< 0.001
	Median	139	137.5	131	134		
	Min - Max	123 - 163	131 - 142	120 - 137	118 - 142		
K	Mean ± SD	4.53 ± 0.71	4.45 ± 0.38	3.67 ± 0.56	4 ± 0.6	4.57	0.01
	Median	4.5	4.5	3.5	4.15		
	Min - Max	2.3 - 7.3	3.9 - 5.2	3.1 - 4.5	3.1 - 4.7		
Cl	Mean ± SD	103.29 ± 4.30	102.96 ± 4.52	88.43 ± 17.23	93.9 ± 15.5	35.85	< 0.001
	Median	104	103	88	101		
	Min - Max	80 - 138	94 - 109	64 - 106	64 - 108		
pH	Mean ± SD	7.39 ± 0.7	7.39 ± 0.06	7.5 ± 0.12	7.47 ± 0.11	8.33	< 0.001
	Median	7.39	7.39	7.51	7.45		
	Min - Max	7.0 - 7.6	7.3 - 7.5	7.3 - 7.7	7.3 - 7.7		
pCO_2_	Mean ± SD	37.35 ± 8.31	36.31 ± 7.77	38.21 ± 8.68	37.96 ± 6.9	0.10	0.89
	Median	36	38.5	40.4	40		
	Min - Max	20.6 - 102.1	23.9 - 46.2	21 - 48	23.9 - 48		
HCO_3_	Mean ± SD	22.17 ± 4.32	22 ± 3.86	30.64 ± 14.58	28.37 ± 12.8	11.98	< 0.001
	Median	21.6	22.3	25.5	25.5		
	Min - Max	9.6 - 43.4	15.6 - 27.6	17.6 - 54	15.6 - 54		

### Consanguinity and family history

Data was available for 816 families. Among 45 families, parents were consanguineous (5.5%). Consanguineous marriages were between the first or second cousins from the descendants of the same generation. There was 1 family with a family history of CF, and 717 families did not have any known history of disease. A history of asthma was reported in 76 families, and atopy or documented allergy was reported in 9 families. Chronic lung diseases, including tuberculosis, were reported in 13 families.

Two out of 17 CF patients had consanguineous parents, and one patient had a family history of chronic lung disease. There was no significant relationship between consanguinity and the diagnosis of CF (P > 0.05).

## Discussion

The aim of this retrospective cohort study was to report our Nanoduct conductivity results and to detail the indications for ascertaining the sweat conductivity test and laboratory values based on results from patients in Turkey. Among the patients who were referred to our pediatric department with various indications for sweat conductivity analysis over a 4-year period, we were able to identify 40 patients with the sweat conductivity results above threshold (> 60 mmol/L). The sweat conductivity test has been approved by the CF Foundation as a screening test at community hospitals [2]. Sweat conductivity may have a role in excluding a diagnosis of CF [2], but sufficient data are not available to recommend the Nanoduct device in diagnosing CF [9]. The consensus committee recommends the following sweat chloride reference ranges for infants up to 6 months: no diagnosis of CF if values are ≤ 29 mmol/L; intermediate if values are in the range of 30 - 59 mmol/L; values are indicative of CF if they are ≥ 60 mmol/L. For infants older than six months the ranges are: ≤ 39, 40 - 59, and ≥ 60 mmol/L, respectively [9].

Conductivity was shown to have approximately the same capacity for distinguishing between CF and non-CF patients. In addition, the sensitivity and specificity of conductivity was similar to results obtained from the Gibson and Cook method in the same subjects [6].

A comparison of the Macroduct and Nanoduct systems were studied in two investigations. In 2005, 111 subjects were tested, and the Nanoduct system was found to be reliable in differentiating CF (mean conductivity, 115 mmol/L; range, 92 to 137) from non-CF patients (mean conductivity, 36 mmol/L; range, 17 to 59) [14]. A second study in 2006 performed 110 tests in 100 patients and concluded that the Nanoduct system cannot be used for the diagnosis of CF due to a high false negative rate [7]. In 2008, Desax et al [5] reported the results of Nanoduct sweat analysis in 1,041 patients; the median conductivity level in non-CF subjects was 37.0 mmol/L (range: 2 - 108 mmol/L), and it was 114.5 mmol/L (range: 60 - 139 mmol/L) for CF patients [5]. Assuming that 59 mmol/L is the upper limit of normal conductivity, the 46 CF patients were correctly diagnosed with a sensitivity of 100% and a specificity of 95.7% [5]. In our study, the median conductivity level in non-CF subjects was 33.0 mmol/L, and the median conductivity level in CF patients was 91 mmol/L (mean ± SD: 88.2 ± 18.7; range: 54 - 119 mmol/L).

Sands et al [15] reported results from 487 Nanoduct conductivity tests, and they demonstrated that CF infants had a mean conductivity of 99.8 ± 18.8 mmol/L, and non-CF infants had mean values of 29.8 ± 7.7 mmol/L. A high correlation between Nanoduct conductivity results and the classic pilocarpine method was found (95% confidence level; r = 0.87) [15]. The optimal cut-off for conductivity tests was 50 mmol/L with a sensitivity of 100% and a specificity of 97.5% [15]. Our results indicated 100% sensitivity and specificity with a cut-off value of 53 in the first conductivity test and 60 with the consecutive test. Although in our study the number of patients confirmed with sweat Cl analysis (n = 20) was relatively smaller than in previous studies, the proposed cut-off values of 50 [15] and 59 [5] were similar to our results (53 and 60) in two consecutive tests with the sensitivity of 100%.

Hammond et al [4] found that CF patients had conductivity values of more than 90 mmol/L, and 430 (91%) non-CF patients had conductivity values of less than 50 mmol/L. They concluded that conductivity was equally satisfactory as chloride measurements [4].

Mishra et al [16] created age-related reference intervals for sweat testing using the Wescor Macroduct System. Sweat chloride increased with age in 282 healthy subjects. The estimated median (95% RI) values for sweat chloride were: 5 to 9 years, 13 mmol/L (1 - 39 mmol/L); 10 to 14 years, 18 mmol/L (3 - 47 mmol/L); 15 to 19 years, 20 mmol/L (3 - 51 mmol/L); and 20 + years 23 mmol/L (5 - 56 mmol/L) [16]. Similarly, we also found positive correlations with age and Nanoduct conductivity results (P < 0.01).

Mastella et al [6] reported specificity and sensitivity of 0.962 at the value of 74 mmol/L for the Macroduct system. Out of 3834 subjects tested with the Macroduct system, the optimal conductivity cut-off value to predict a positive CF diagnosis was ≥ 90 mmol/L, with 99.66% sensitivity and 100% specificity [17]. The optimal value to predict a negative CF diagnosis was < 75 mmol/L, with 99.25% sensitivity and 93.37% specificity [17].

Sweat testing performed at 40 and 60 days of age in 1003 infants revealed no correlation between age and sweat chloride concentration [18]. Heeley et al [3] compared the conductivity results of CF patients with the non-CF control group. Their control group consisted of 154 children with symptoms of a failure to thrive, gastrointestinal obstruction, diarrhea, respiratory problems, and jaundice. The range of conductivity was 18 - 71 mmol (NaCl eq)/L in the control group, whereas it was 67 - 141 mmol (NaCl eq)/L in the CF group. Older children (9 - 15 years) in the control group showed increased sweat test results that overlapped with those of CF patients [3].

The mean age at diagnosis of CF was 52.7 months in India [19] and 2.88 ± 3.5 years in Saudi Arabia [20], whereas the mean age at onset of symptoms was 10.7 ± 19.5 months [19]. The mean age at the time of diagnosis in Turkey was 31.2 ± 41.4 (range: 1.5 - 168) months [12]. In our study, the mean age at diagnosis was 13.8 ± 23.7 months with a median of 4 months. The duration of time between the onset of symptoms and the diagnosis of CF illustrates the need for an educational program for medical staff about CF.

Bar-Zohar et al [21] retrospectively studied 255 children who were referred for sweat testing from 1991 - 1996 [21]. The prevalence of asthma was 36.5%, and the prevalence of the failure to thrive was 7.9% [21]. The prevalence of asthma and the failure to thrive in our cohort was 5.6% and 4%, respectively.

Hyponatremia, hypochloremia and metabolic alkalosis are well recognized in CF patients. These can occur both as a presentation or a complication of CF [22]. Pseudo- Bartter Syndrome in children with CF should be identified early to avoid recurrent attacks and high morbidity. The prevalence of pseudo-Bartter Syndrome was reported to be between 12% [23] and17.6% [24] in Turkey. The mean ± SD values of electrolytes and arterial blood gas in CF patients were as follows: plasma sodium, 126.1 ± 6.0; potassium, 3.1 ± 0.7; chloride, 77.8 ± 19.4; blood pH, 7.6 ± 0.6; and bicarbonate, 38.9 ± 5.5 mmol/L [24]. Yalc?n et al [23] found similar mean values of plasma sodium, potassium and chloride: 126, 3.1 and 79 mmol/L, respectively. The mean blood pH was 7.53, and the mean bicarbonate was 35 mmol/L [23]. In our study, the mean ± SD values of electrolytes and arterial blood gas in CF patients were as follows: plasma sodium, 133.43 ± 6.2; potassium, 4 ± 0.6; chloride, 93.9 ± 15.5; blood pH, 7.47 ± 0.11; and bicarbonate 28.37 ± 12.8 mmol/L.

The incidence of metabolic alkalosis with hypoelectrolytemia was 16.5% in an infant CF population in Macedonia [25] and 16.2% in Spain [22]. Metabolic alkalosis with low electrolytes as the presentation of CF was 16.8% in 77 CF patients between the ages of 3 and 14 months [26]. Ozcelik et al [27] reported a sodium chloride deficiency in 12 of 46 CF patients who had mean plasma sodium, potassium and chloride levels of 122.9 (range 106 - 135), 2.5 (range 1.6 - 3.5), and 73.3 (range 60 - 90) mEq/L, respectively [27]. Unexplained hypochloremic metabolic alkalosis should always be considered in the diagnosis of CF. We believe that metabolic alkalosis with hypoelectrolytemia is suggestive of CF and should be referred for sweat testing.

Consanguinity was not found to be major factor contributing to the incidence of CF in Turkey [10]. The highest genetic heterogeneity in a population with respect to CF was found, and a minimum carrier frequency of 1 in 50 was assessed in the Turkish population with under-diagnosed CF [10]. In our study, there were 45 consanguineous marriages, but we did not find any significant relationship between consanguinity and the diagnosis of CF in our patients.

Unfortunately, our study had several limitations. Data for arterial blood gas and electrolytes were not available in all cases because of the retrospective design. Some of our patients might have been treated later at another hospital for recurrent symptoms, so we may not know the true percentage of patients with CF. Most of the data in the literature comes from highly qualified CF centers, and there is not enough data from non-specialized clinical settings. The purpose of this study was to report the ways in which we attempt to deal with the high numbers of patients with suspected CF, without enough pediatric pulmonologists and qualified CF centers. Data reported in the present work provide support for the need of neonatal screening programs in developing countries with a high incidence of CF carriers.
